# A Quadrature Single Side-Band Mixer with Passive Negative Resistance in Software-Defined Frequency Synthesizer

**DOI:** 10.3390/s18103455

**Published:** 2018-10-14

**Authors:** Dongsheng Liu, Ang Hu, Kefeng Zhang

**Affiliations:** School of Optical and Electronic Information, Huazhong University of Science and Technology, 1037 Luoyu Road, Wuhan 430074, China; zhangkefeng@hust.edu.cn

**Keywords:** quadrature single side-band mixer, wireless sensor networks (WSN), frequency synthesizer (FS), phase locked loop (PLL), software-defined radio (SDR)

## Abstract

Software-defined radio (SDR) is a good solution for complying with the existing and incoming protocols for emerging wireless sensor networks (WSN) and internet of things (IoT) applications. The frequency synthesizer in a SDR tranceiver usually consists of a phase locked loop (PLL) and a post synthesizer. The PLL is the narrow band signal source and the post synthesizer generates wideband outputs by mixing and dividing. Compared with a frequency synthesizer utilizing the wideband PLL, this synthesizer features relatively constant loop parameters and mitigates the requirement for the oscillator. In this paper, a quadrature single side-band (QSSB) mixer with the proposed passive negative resistance (PNR) for frequency mixing in a post synthesizer is presented. The PNR is achieved by biasing the Metal-Oxide-Semiconductor Field-Effect Transistors (MOSFET) of the cross-coupled pair at the deep-triode region periodically and incorporates an inductor and a cap-array as the mixer load. Compared with the traditional single side-band mixers utilizing Inductor-Capacitor (LC) resonant loads or quality factor enhanced (Q-enhanced) LC resonant loads, which suffer from a selectivity versus working range trade-off, the mixer employing the proposed loading structure provides not only a wide operating range, but also a superior image side-band rejection ratio (ISRR). Moreover, the oscillating risk in conventional mixers adopting Q-enhanced LC resonant loads is eliminated. A wideband frequency synthesizer employing the proposed mixer was implemented in a TSMC 0.18 µm CMOS process and the mixer performed ISRR of 40–57 dB and 30–57 dB across 2.5–3 GHz and 2.3–3.2 GHz, respectively. The power consumption of the QSSB mixer, including buffer, is 18 mA from a 1.8 V supply and the active area is 0.445 mm^2^. The measurement results provide validation that the proposed QSSB mixer is suitable for wideband software-defined frequency synthesizers and other frequency generating systems.

## 1. Introduction

**Figure 1:**
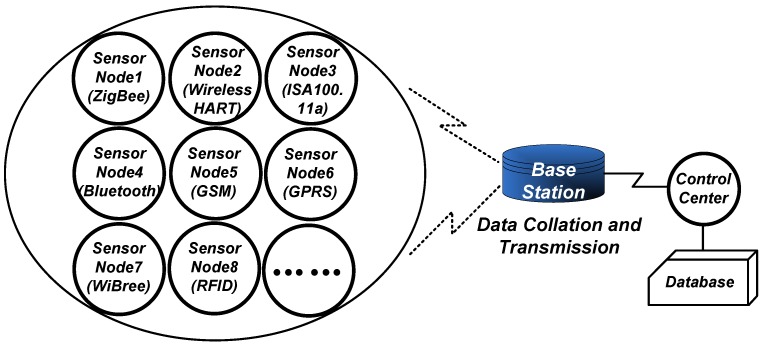
A typical wireless sensor network (WSN) topology.

The wireless sensor network is an enabling technology of the internet of things and has emerged in recent decades. A complete wireless sensor network (WSN) system consists of many randomly deployed sensor nodes, a base station (or sink), a control center and a database, as illustrated in Figure 1. Various types of sensor nodes gather information about physical objects or environmental conditions, including the surrounding temperature, humidity, moisture, water flow and so on. The base station controls these sensor nodes and acts as a gateway to transmit the gathered data back to the control center. Although the use of sensor nodes in a WSN is currently limited to the fixed industrial-scientific-medical (ISM) band, they are perfectly able to conform with the developing trend of the internet of things (IoT) where any sensor node can communicate with the base station employing any wireless protocol. For instance, in a target monitoring field, it would be meaningful if sensor node 1 and sensor node 4 could utilize ZigBee and Bluetooth protocols separately to make contact with the base station, as presented in Figure 1. As WSN and IoT markets are growing rapidly, this multiple-communication-techniques scenario will be ubiquitous in the future.

Until now, many wireless protocols have been used in WSNs and IoT systems including standard mobile telephony (GSM, GPRS), broadband techniques (802.11 a/b/g) and wireless personal area networks (ZigBee, IEEE 802.15.4, Bluetooth,). In addition, many other wireless protocols are springing up, such as the fifth generation (5G), which operates at 3.3–3.6/4.8–5 GHz in China. Some typical protocols with their corresponding main characteristics, covering operating frequencies ranging from 13.56 MHz to 2.4 GHz, are summarized in Table 1. As the bridge connecting the sensor nodes and the control center, the performance of the base station is the bottleneck of the system. To comply with as many protocols as possible, a base station grounded on software-defined radio is a good solution [1], but one which puts stringent requirements on having a frequency synthesizer (FS) with a wider frequency range, and lower phase noise and spurs.

Generally, the FS in software-defined radio (SDR) is composed of a narrow band phase locked loop (PLL) and post synthesis circuit [2,3,4,5,6,7,8,9,10,11,12]. Compared with a wideband PLL employing multiple voltage controlled oscillator (VCO) cores, the PLL in this FS structure characterizes relatively constant loop parameters and releases the requirement for the oscillator. The post synthesis circuit is fed by the PLL outputs and generates wideband outputs by mixing and dividing. For Most transceivers utilize in-phase and quadrature local oscillator (LO) signals to suppress image signals and support advanced quadrature amplitude modulation. A quadrature single side-band (QSSB) mixer is usually adopted in a post synthesis circuit to produce quadrature outputs.

The widely employed QSSB mixers are based on the double-balanced Gilbert structure which features sufficient LO leakage suppression at the output. Moreover, to obtain purity of LO signals, the image side-band signals should also be attenuated. The LC resonant loads are widely utilized in the QSSB mixer to pick out the desired signals [3,6,13,14]. However, the working range and the selectivity conflict with each other. The Q-enhanced LC resonant loads are utilized in the QSSB mixer [5,7,15,16] for better selectivity characteristics, whereas the risk of oscillating is increased. Furthermore, if the mixer starts oscillating, the working range will be limited, because of the injection locked phenomenon, and the phase noise will deteriorate.

In order to release the contradiction between selectivity and working range and reduce the oscillating risk, a QSSB mixer adopting a novel cross-coupled pair is proposed. The cross-coupled pair performs positive resistance characteristics in small output voltage conditions and provides negative resistance in large output conditions. Contrary to the conventional negative-Gm cross-coupled pair in VCO, which performs negative resistance all the time, the proposed structure provides negative resistance under a specified condition, thus reducing the oscillating risk. We name this negative resistance passive negative resistance (PNR). As long as the cross-coupled pair performs the PNR characteristic, the mixer selectivity is swiftly enhanced, because of the positive feedback, and finally reaches a balance due to the negative feedback. Implemented in TSMC 0.18 µm CMOS process, the proposed mixer performs image side-band rejection ratios (ISRR) of 40–57 dB and 30–57 dB across 2.5–3 GHz and 2.3–3.2 GHz respectively, while consuming 18 mA. The measurement results demonstrate the mixer with PNR can provide both wide working range and good selectivity simultaneously. Moreover, the mixer is absolutely stable and the oscillating risk in the conventional mixer employing Q-enhanced resonant loads is eliminated. This QSSB mixer is suitable for wideband software- defined frequency synthesizer and other frequency generating systems.

This paper is organized as follows: The working principle of QSSB mixer is introduced in Section 2 and the proposed QSSB mixer with PNR is provided in Section 3. The measurement results and discussions are presented in Section 4. Finally, conclusions are presented in Section 5.

## 2. Quadrature Single Side-Band Mixer

**Figure 2:**
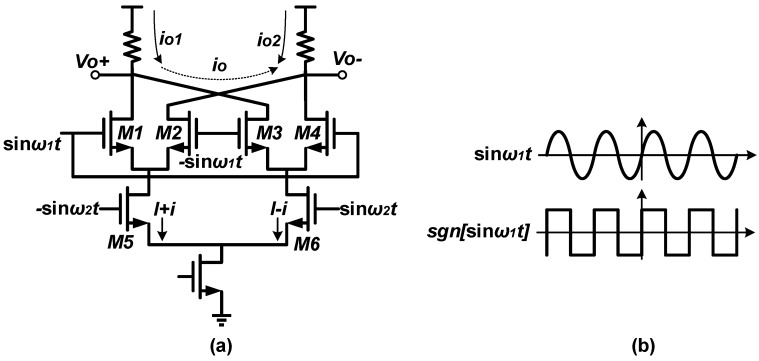
(**a**) Typical double-balanced active mixer; (**b**) characteristics of the switching signal.

Mixers can be divided into active mixers and passive mixers. The former feature good isolation and better gain, while the latter are characterized by superior linearity. Because of the unacceptable switch loss and gain attenuation of passive mixers at high frequencies, the mixers utilized in FS are usually based on active mixers. A typical double-balanced active mixer is presented in Figure 2a. The transistors *M1*–*M4* work as switches, and *M5*–*M6* work as a transconductance pair. If the amplitude of the LO signal is large enough, *M1*–*M4* can be regarded as ideal switches. When the LO signal sin*ω*_1_*t* is in positive cycle, *M1*/*M4* turn on and *M2*/*M3* turn off, yielding a small-signal differential output current of 2*i*. Conversely, the output current is −2*i* when the LO signal is in negative cycle. In other words, the output current direction switches periodically according to the polarity of the LO signal sin*ω*_1_*t*. Therefore, the differential output current can be written as

(1) $$i_{o}=2i\times sgn[\sin\omega_{1}t]\;$$

The characteristics of function $sgn\left[\sin\omega_{1}t\right]$ are illustrated in Figure 2b. As $sgn\left[\sin\omega_{1}t\right]$ is an odd function, the Fourier series contains only sinusoid components and is given by

(2) $$sgn\left[\sin\omega_{1}\right]=\sum_{n=1}^{\infty }b_{n}\sin(n\omega_{1}t)\;$$

where $b_{n}=\frac{2}{T}\left(\int_{0}^{\frac{T}{2}}\sin n\omega_{1}tdt-\int_{\frac{T}{2}}^{T}\sin n\omega_{1}tdt\right)=\frac{2\left(1-\cos n\pi \right)}{n\pi }$. For the small-signal current 2i can be written as $-2*g_{m}\;\sin\omega_{2}t$, the output current $i_{o}$ can be represented as

(3) $$i_{o}=-2g_{m}\sum_{n=1}^{\infty }b_{n}\sin\omega_{2}t\times \sin n\omega_{1}t\;$$

**Figure 3:**
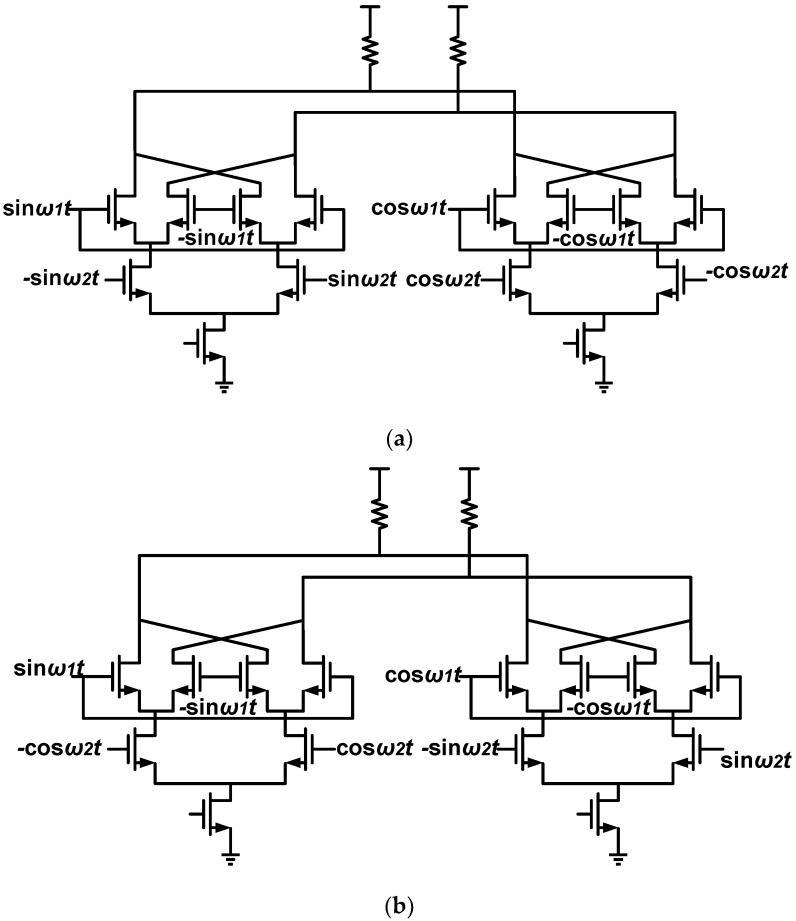
(**a**) The I-branch single side-band mixer; (**b**) the Q-branch single side-band mixer.

It can be observed that the output current contains double side-band components such as *ω*_2_ – *ω*_1_, *ω*_2_ + *ω*_1_ and so on. A single side-band (SSB) mixer is composed of two double-balanced mixers that share the same loads, as presented in Figure 3a. The output current of the left mixer is given in Equation (3) and that of the right mixer is $2g_{m}*\sum_{n=1}^{\infty }a_{n}\cos\omega_{2}t*\cos n\omega_{1}t$,so the total output current is

(4) $$i_{out}=2g_{m}\overset{\infty }{\underset{n=1}{\sum }}k_{n}(\cos\omega_{2}t\times \cos n\omega_{1}t-\sin\omega_{2}t\times \sin n\omega_{1}t)=2g_{m}\overset{\infty }{\underset{n=1}{\sum }}k_{n}\cos\left(\omega_{2}t+n\omega_{1}t\right)\;$$

where we assume that the circuit is symmetrical and the Fourier coefficient $a_{n}$ is equal with $b_{n}$ and represented as $k_{n}$. Therefore, the output current contains only the upper side-band components $\omega_{2}+n\omega_{1}$ and the lower side-band signals are suppressed. 

A QSSB mixer is made up of two identical SSB mixers with different input signal sequences. If we name the mixer in Figure 3a the I-branch SSB mixer, the Q-branch SSB mixer can be presented in Figure 3b with an output current of

(5) $$i_{out}{}^{\prime }=-2g_{m}\overset{\infty }{\underset{n=1}{\sum }}k_{n}(\cos\omega_{2}t\times \sin n\omega_{1}t+\sin\omega_{2}t\times \cos n\omega_{1}t)=-2g_{m}\overset{\infty }{\underset{n=1}{\sum }}k_{n}\sin\left(\omega_{2}t+n\omega_{1}t\right)\;$$

By multiplying the output current in Equations (4) and (5) with relative loads, the quadrature signals can be obtained. In reality, the mismatches of the input signals, the circuit asymmetry and the nonlinearity can result in image side-band signals. It is necessary for the loads to have the ability to pick out the desired signals and suppress the image side-band signals.

**Figure 4:**
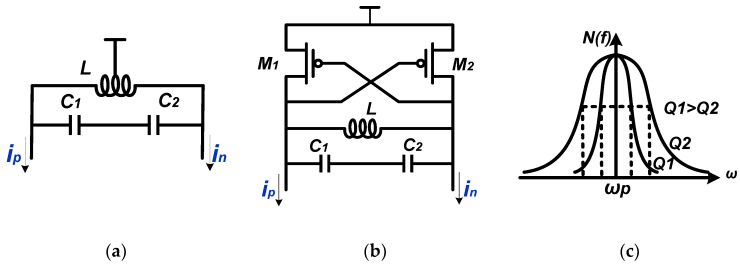
(**a**) The LC resonant load; (**b**) The Q-enhanced LC resonant load; (**c**) The tuning curve of the LC resonator.

The LC resonant loads and the Q-enhanced LC resonant loads are widely used in SSB mixers and are presented in Figure 4a,b separately. The cross-coupled negative-Gm pair used in conventional LC VCO is employed for a higher quality factor (Q) and better selectivity. The main drawback of the two kinds of LC loads is the conflict between selectivity and working range, as depicted in Figure 4c. A LC resonant load with higher Q performs better selectivity, while the working range is narrower than those with lower Q. Meanwhile, a mixer utilizing the Q-enhanced LC resonant load has the risk of oscillating, which could reduce the working range and degrade phase noise performance because of the injection locked phenomenon.

## 3. Proposed PNR and QSSB Mixer

### 3.1. The Proposed PNR

**Figure 5:**
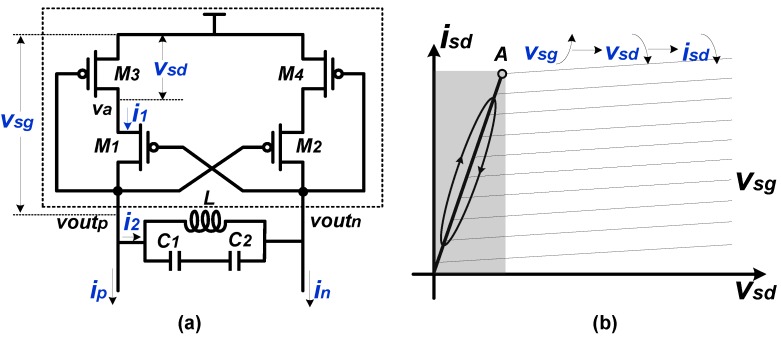
(**a**) The proposed LC resonant loads with passive negative resistance (PNR); (**b**) The I-V curve of *M3*/*M4*.

**Figure 6:**
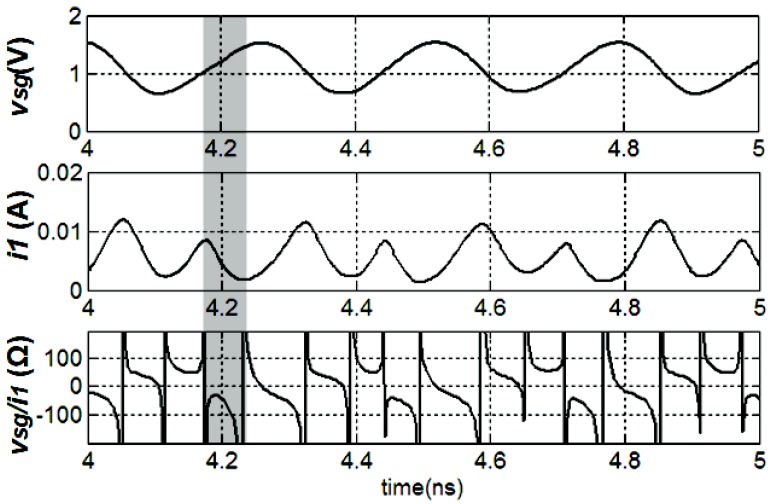
The transient simulation results of $v_{sg}$, $i_{1}$ and output resistance.

In order to release the contradiction between the selectivity and the working range of the conventional LC resonant load, we proposed a novel LC resonant load. The proposed load structure is illustrated in Figure 5a. Given that the circuit is symmetrical, we only analyze the half circuit for simplicity. If the amplitude of the output signal $vout_{p}$ is small and the circuit is biased properly, the *M3* works at the triode region or the saturate region. As the amplitude increases, or as $v_{sg}$ increases, the *M3* could be forced to work at the deep-triode region periodically. In this deep-triode region, with $vout_{p}$ decreasing, the $v_{sg}$ and$\;v_{a}$ increase accordingly, yielding a decreased source-to-drain voltage $v_{sd}$. As illustrated in the gray region in Figure 5b, the decreased $v_{sd}$ is accompanied by the decreased $i_{sd}$. In other words, with $v_{sg}$ increasing, the source-to-drain current $i_{1}$ decreases, resulting in a negative resistance. The transient simulation results of $v_{sg}$, $i_{1}$, and $v_{sg}/i_{1}$ are presented in Figure 6, and the negative resistance is depicted in the gray region. This negative resistance is performed only when the output amplitude is large enough and would not be presented if the output amplitude is small. Compared to a mixer utilizing a conventional negative-Gm pair, a mixer adopting the proposed PNR-based load can reduce the risk of oscillation.

**Figure 7:**
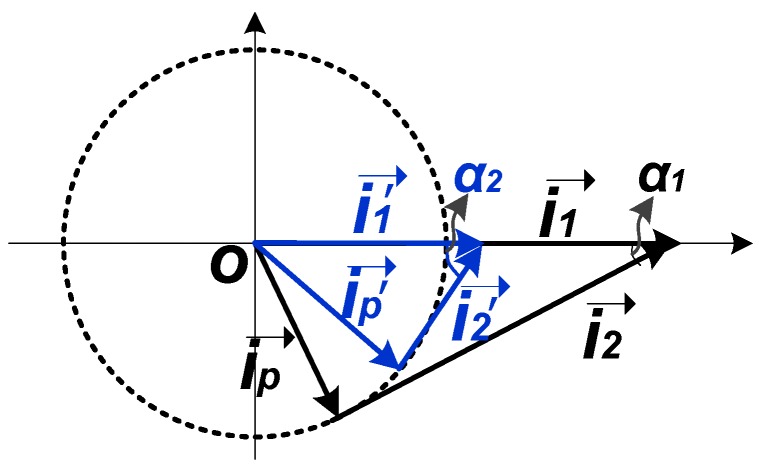
The vector interpretation of current changing.

As long as the PNR is performed, the quality factor of the proposed load structure increases, resulting in a larger output impedance. For a mixer that is biased with a current source, the current $i_{p}$ in Figure 5a is usually fixed, and the amplitudes of $vout_{p}$ and $vout_{n}$ would increase as impedance increases, which in turn compels *M3*/*M4* to work in the deep-triode region for a long time and produces larger than average PNR. This is a positive feedback that makes the output amplitude become larger and larger. Because *M3* works in the deep-triode region for a long time and the average value of $i_{1}$ keeps decreasing, the amplitude of $i_{2}$ has to be decreased to maintain the vector relationship between $i_{p}$, $i_{1}$ and $i_{2}$, as illustrated in Figure 7. When PNR is not performed and $i_{1}$ is relatively large, the current $i_{2}$ is also large. Once PNR is performed, accompanied by a decreased average current $\vec{i_{1}{}^{\prime }}$, the current vector across the LC tank switches from $\vec{i_{2}}$ to $\vec{i_{2}{}^{\prime }}$ with a smaller amplitude. This is a negative feedback that maintains the balance of the output amplitude. In conclusion, the positive feedback produces large negative resistance and ensures high Q for superior selectivity, while the negative feedback guarantees the balance of the circuit.

### 3.2. The Proposed QSSB Mixer

**Figure 8:**
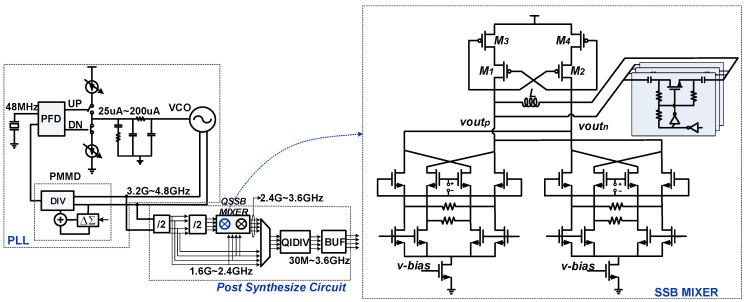
The proposed frequency synthesizer (FS) and single side-band (SSB) mixer.

The proposed QSSB mixer in a wideband FS is presented in Figure 8. The FS is composed of a PLL and post synthesis circuit. The PLL is made up of a phase frequency detector (PFD), a charge pump (CP), an off-chip loop filter, an integrated VCO and a programmable multi-modulus divider (PMMD) and provides the differential outputs ranging from 3.2 to 4.8 GHz. The post synthesis circuit consists of the divide-by-2 dividers, the QSSB mixer, and the quadrature divider (QIDIV) and is fed by the differential VCO outputs. The first divide-by-2 divider is based on the conventional current-mode logic (CML) structure and gives the 1.6–2.4 GHz quadrature outputs, while the second one is a regenerative divider and realizes the function of the quadrature-in quadrature-out (QIQO). The outputs of the two dividers are delivered to the QSSB mixer to produce the 2.4–3.6 GHz quadrature outputs. By combining the two quadrature signal sources, the 1.6–3.6 GHz quadrature outputs are obtained. By sending the quadrature outputs into the QIDIV, that consists of 6 stages of divide-by-2 dividers, the output frequencies ranging from 30 M to 3.6 GHz are covered. Compared with a PLL utilizing multiple VCO cores and a set of dividers to produce the wideband outputs, the PLL in the proposed FS adopts only one VCO core with differential outputs covering 3.2–4.8 GHz, which reduces the loop parameters variation and is beneficial for optimization. Moreover, the PMMD in the PLL could work at a lower frequency and consume less power.

**Figure 9:**
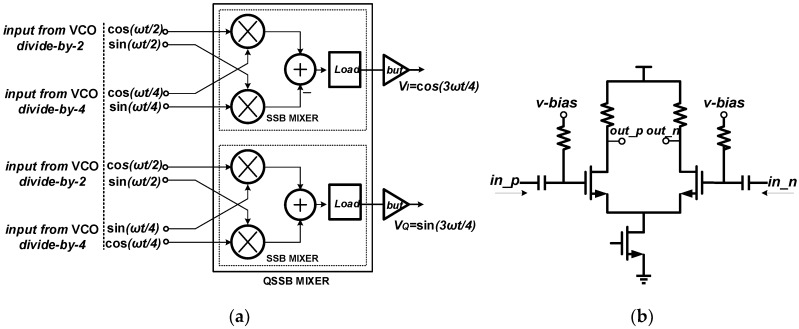
(**a**) The overall quadrature single side-band (QSSB) mixer structure; (**b**) the output buffer.

The QSSB mixer is composed of two identical SSB mixers and two output buffers. A 3-bit cap array is utilized to extend the operating range. Meanwhile, the source degeneration technique in LO ports is employed to further improve the linearity, splitting the RF ports into 8 devices. The QSSB mixer structure is presented in Figure 9a, with two input frequencies at ω/2 and ω/4 and an ideal output frequency at 3ω/4, where ω represents the VCO oscillating frequency. To avoid loading effects and provide better drive capability, output buffers are employed and the structure is presented in Figure 9b.

The transient output waves, the operating range and the ISRR of a QSSB mixer employing the conventional LC resonant load, the Q-enhanced LC resonant load, and the proposed load are simulated separately for comparison. The Q-enhanced LC resonant load is similar to the proposed one in Figure 5 except *M3*/*M4*, and the conventional LC resonant load excludes *M1*–*M4*. When biased with the fixed current source, the power consumptions of the mixers adopting different loads are the same. The simulation is based on the post synthesizer, fed by the ideal differential VCO outputs.

**Figure 10:**
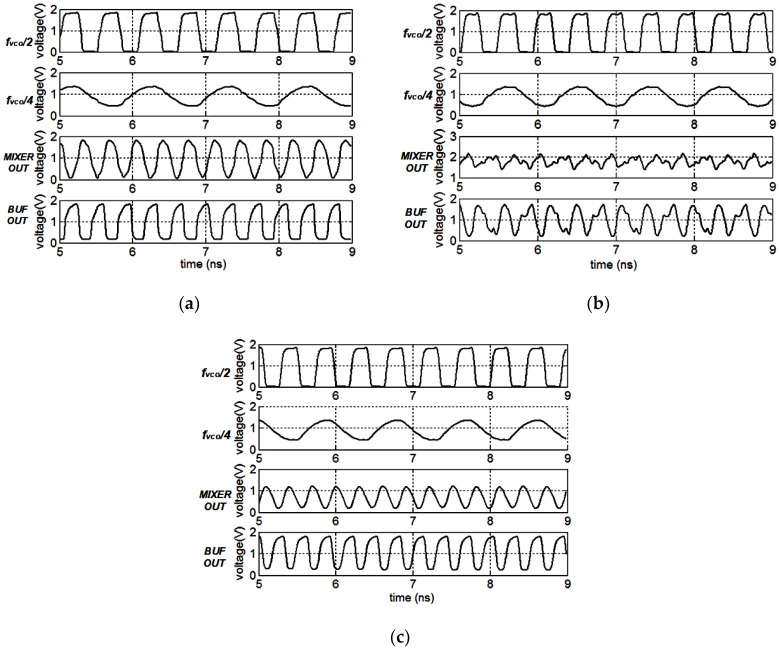
The transient simulation results of the QSSB mixer employing: (**a**) the Q-enhanced LC resonant load; (**b**) the conventional LC resonant load; (**c**) the proposed load.

The transient simulation results of the mixer with fixed cap array control signals and different types of loads are presented in Figure 10a–c. The input frequency is set to be 4.4 GHz and the ideal output frequency is 3.3 GHz. The peak-to-peak voltage of the mixer outputs reach 1.8 V when the mixer adopts the Q-enhanced LC resonant loads, as presented in Figure 10a. After passing through the following buffer, the output amplitude does not appear to change. In fact, the mixer is injection locked under this condition. When employing the conventional LC resonant load, the mixer produces unclean signals because of the limited selectivity, and the transient outputs are presented in Figure 10b. The pure output signals are provided at the mixer output with an amplitude of about 0.5 V when utilizing the proposed load, as depicted in Figure 10c.

**Figure 11:**
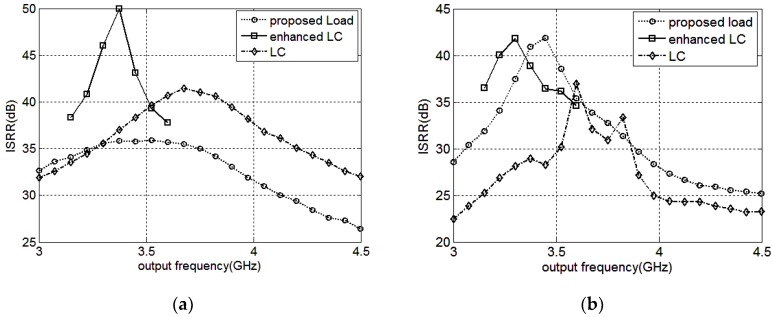
The image side-band rejection ratio (ISRR) versus the operating range at the mixer outputs (**a**); and buffer outputs (**b**).

The ISRR versus the operating range at the mixer’s outputs and the buffer’s outputs are presented in Figure 11a,b respectively. For the two inputs ω/2 and ω/4,and the desired output locates at 3ω/4, the image side-band is ω/4, the same as one of the inputs. Thus, the input signals could degrade the ISRR directly by feedthrough or other side-effects. When employing the Q-enhanced LC resonant load, the mixer provides the best ISRR performance at the mixer’s outputs with the maximum value of about 50 dB at 3.375 GHz. However, the output frequency range is 3.15 G–3.6 GHz because of the injection locked phenomenon. The mixers adopting the LC resonant load and the proposed load perform over a wider operating range, and the ISRR performance of the former is superior for the absent utilization of nonlinearity components in the LC load. In reality, at the outputs of the mixer employing the LC resonant load, apart from the image side-band signal, some other side-band signals also exist because of the low Q LC tank, leading to the unclear transient outputs as presented in Figure 10b. The ISRR simulation results at the mixer’s outputs are presented in Figure 11a. At the buffer’s outputs, the ISRR of the mixer utilizing the LC or Q-enhanced LC resonant load deteriorates and that of the mixer adopting the proposed load improves from 3.2 G–3.6 GHz; the simulation results are depicted in Figure 11b. Compared with the mixer employing the conventional loads, the proposed one, adopting the PNR-based load, performs over a wider operating range and has a higher ISRR simultaneously.

There are two reasons why the ISRR changes at the buffer output. The first one is the different conversion gains at different side-bands. The signal power at the image side-band is smaller than that at the desired side-band, and the latter would saturate the output buffer, resulting in a small gain. For example, the mixer’s output amplitude reaches 0.9 V as presented in Figure 10a. After being amplified by the buffer, the amplitude is still about 0.9 V, indicating that the desired signal is amplified to a limited extent, whereas the signals at the image side-band would be amplified, thus degrading the ISRR at the buffer’s output. The second one is the nonlinearity effects. The buffer would work in the nonlinear region if the input signal is large, producing many harmonic tones. These tones could locate at the same frequency with different phase and amplitude and could be superimposed or cancel each other out. For instance, if the tone at the image side-band is enhanced while that at the desired side-band is suppressed, the ISRR deteriorates. This is what happens to the mixer that enriches harmonic tones at the mixer core outputs. As for the mixer adopting the proposed load, the desired side-band would be amplified properly because of fewer harmonic productions at the mixer outputs.

**Figure 12:**
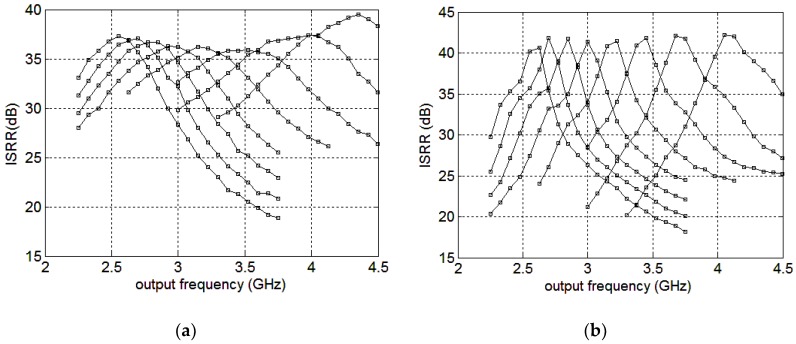
The overall ISRR versus the output frequency at the mixer outputs(**a**); and the buffer outputs (**b**).

The overall ISRR simulation results at the mixer’s outputs and the buffer’s outputs by tuning the mixer cap-array are presented separately in Figure 12a,b. The output frequency range covers 2.25–4.5 GHz. At the mixer core outputs, the ISRR is 33–39.5 dB, and is 30–42 dB at the buffer outputs. In 2.4–3.6 GHz, the ISRR are 35.8–37.3 dB and 35.2–41.8 dB at the mixer core outputs and the buffer outputs, respectively.

## 4. Measurement Results and Discussion

**Figure 13:**
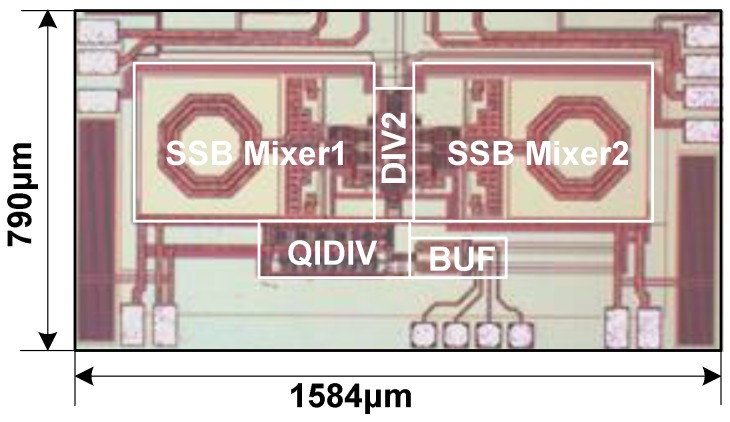
Micrograph of the proposed post synthesis circuit.

The proposed QSSB mixer and the frequency synthesizer are implemented in a TSMC 180 nm CMOS RF process, and the micrograph of the post synthesis circuit is presented in Figure 13 with an area of 1.25 mm^2^. This process provides one poly layer and six metal layers, with a 4.6 μm ultra-thick top metal (*M6*) and 8.15 μm distance between *M6* and the substrate. The permittivity, thickness and resistivity of the substrate are 11.9, 250 μm and 10 Ωcm, separately. Because of the adopted LC tank in the mixer core, most of the area is made up of the inductors and the total active area of the QSSB mixer is about 0.445 mm². The layout of the QSSB mixer is designed as symmetrically as possible for better side-band rejection. The output spectrum and phase noise are measured by Rohde Schwarz FSV7 signal analyzer. The ISRR is measured through the output spectrum by calculating the power level discrepancy between the desired signal and the image side-band signal. In this condition, the FS is unnecessarily locked, and the output frequency is switched by controlling the VCO cap array. By changing the QSSB mixer cap array, the eight frequency-ISRR curves are obtained. The phase noise is measured using the signal analyzer directly in the locked state.

**Figure 14:**
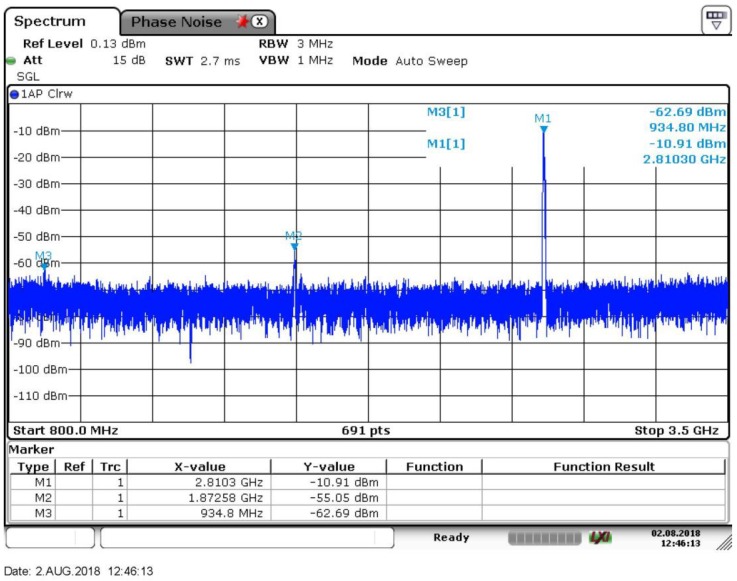
Measured output spectrum at 2810 MHz.

**Figure 15:**
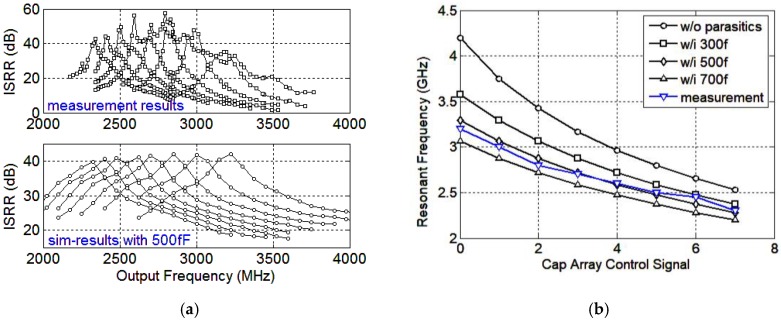
(**a**) ISRR measurement and simulation results versus the output frequency; (**b**) resonant frequencies versus the mixer cap array control signals.

The output spectrum is presented in Figure 14. The two input signals are at 1872 MHz and 936 MHz with the power level of −55 dBm and −62.7 dBm, respectively. The desired tone is at 2808 MHz and the signal power is about −10.9 dBm, producing an ISRR of 51.8 dB. The total ISRR measurement results against the output frequency and the ISRR simulation results, with 500 fF parasitic capacitance, are presented in Figure 15a. It can be observed that the measured operating frequencies are smaller than those of the simulation results presented in Figure 12b, mainly due to the parasitic capacitors decreasing the LC resonating frequencies. When including the 500 fF parasitic capacitors, the resonating frequency simulation results are in accordance with the measurement results. The simulated and measurement resonant frequencies versus the mixer cap-array control signals are presented in Figure 15b. When the parasitic capacitance is set at 500 fF, the simulation results are consistent with the measurement results as shown in Figure 15a. The peak ISRR measurement result is about 57 dB and is larger than that of the simulation result. Furthermore, the measurement operating bandwidth of each curve is much smaller than the simulation result. Considering the conflict between Q and bandwidth, the measurement results imply that the measured quality factor of the implemented load is much higher than the simulation result. The ISRR performances are 40–57 dB and 30–57 dB across 2.5–3 GHz and 2.3–3.2 GHz, respectively. From 3–3.6 GHz, the ISRR drops off drastically, partially because the inverter buffer attenuates the high frequency signals. The inverter buffer delivers −5~0 dBm output power at 30 M–3 GHz while providing −5~−15 dBm at 3–3.6 GHz, implying the buffer attenuates 5–10 dB at 3–3.6 GHz. If the outputs locate at 3–3.6 GHz, the image side-band signals locate at 1–1.2 GHz. In this condition, the desired outputs are attenuated while the image side-band signals are not suppressed to the same extent, leading to the drop-off in the ISRR characteristic. In addition, the gain mismatch and phase imbalance of the VCO outputs can also degrade the ISRR performance. By optimizing the inverter buffer and compensating the gain and phase mismatch of the VCO outputs, the ISRR performance could be improved.

**Figure 16:**
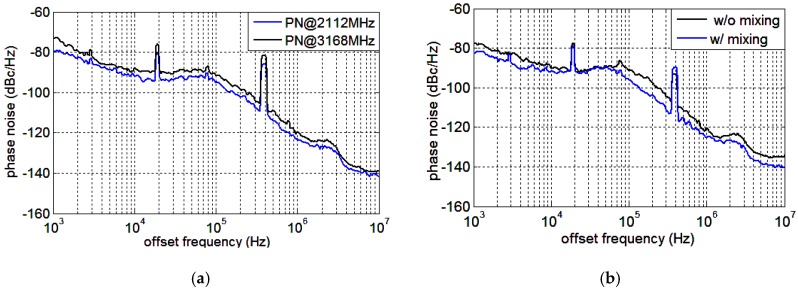
Phase noise measurement results: (**a**) at the input 2112 MHz and output 3168 MHz; (**b**) the same frequency produced by PLL directly or by mixer indirectly at 2376 MHz.

The phase noise measurement results at 2112 MHz and 3168 MHz are presented in Figure 16a, and the spot phase noise at 10 KHz and 1 MHz offsets are −92, −88 and −123, −120 dBc/Hz, respectively. The phase noise distinctions between 2112 MHz and 3168 MHz from 1 KHz to 10 MHz offsets are 3–4 dB. The output frequency 3168 MHz is produced by mixing 2112 MHz and its divide-by-2 output 1056 MHz. Therefore, the two signals originate from the same core synthesizer signal and the phase noise deviation is mainly caused by the QSSB mixer. Ideally, as frequency multiplied by a factor of 1.5, the phase noise would increase about 20log1.5≈3.5 dB, which is in accordance with the measurement results, indicating the noise contributed by the mixer is negligible. The spurs at 20 KHz and 400 KHz are caused by the interfering signals at the input. By employing a pure reference signal, these spurs can be eliminated. The desired VCO tuning range is 3.2–4.8 GHz. In fact, the measurement tuning range of the VCO is 2.5–5 GHz for an efficient frequency margin. Therefore, the target output frequency can be produced by mixing indirectly or by PLL directly. The phase noise measurement results at 2376 MHz, with or without the mixing process, are illustrated in Figure 16b. When directly output, the VCO oscillates at 4752 MHz and the target frequency is obtained by divide-by-2 operation. Otherwise, the VCO works at 3168 MHz and the desired frequency is generated by mixing. As can be seen, the phase noise of the outputs given indirectly is superior to that given directly. In fact, the phase noise of the core synthesizer signal at 3168 MHz is superior to that at 4752 MHz for the lower operating frequency. Although the mixer induces some noise, the output signal originating from 3168 MHz is purer than that from 4752 MHz. The QSSB mixer provides another option for low phase noise frequency synthesizer design.

**Figure 17:**
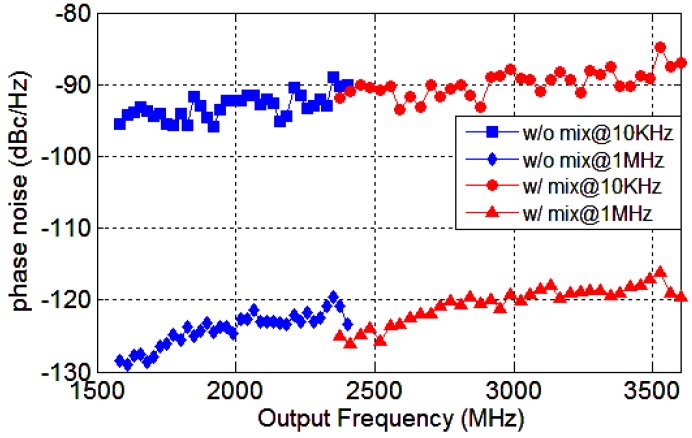
Summarized phase noise performance from 1.6 to 3.6 GHz.

The overall phase noise performances from 1.6 GHz to 2.4 GHz and 2.4 GHz to 3.6 GHz are summarized in Figure 17. The 2.4–3.6 GHz output frequencies are the mixing products of 1.6–2.4 GHz. The spot phase noise at 10 KHz and 1 MHz offsets are less than −87 dBc/Hz and −118 dBc/Hz respectively. Around 2.4 GHz, it can be observed that the phase noise of the outputs without mixing are inferior to those with mixing. This is in accordance with the former discussion. With output frequency decreasing, the phase noise decreases accordingly and the noise of the outputs without mixing are superior to those with mixing for the lower carry frequencies. The performance comparison between this work and the references is presented in Table 2. The frequency synthesizers adopting SSB mixers are mostly for Ultra Wide Band (UWB) application and care about the ISRR at discrete frequencies, such as in References [8,9,17,18,19,20,21,22,23,24], so performance can be optimized by tuning the capacitor array. The ISRR of the SSB mixer in Reference [6] is about 42 dB in the continuous range of 5–6 GHz by utilizing harmonic rejection technique. If employing this technique, the signals *v(t)*, *v(t + T/4)* and *v(t − T/4)* are required to cancel the third- and the fifth-order harmonics and obtain a better ISRR. However, the output frequencies are 5/4 times larger than the input frequencies, which is not adequate for some applications. For the proposed PNR which can improve the selectivity of the LC resonant loads, the mixer employing the PNR-based load performs better ISRR than the mixer adopting the LC loads. The oscillating risk of the conventional mixer utilizing the Q-enhanced LC resonant loads is also eliminated. Compared to these earlier works, the proposed QSSB mixer can provide superior ISRR in a wideband continuous output frequency range. However, the proposed load presents a PNR characteristic when the output amplitude is large enough, implying that the mixer consumes a lot of power. By optimizing the bias point of the PNR, power consumption would be improved. This QSSB mixer is suitable for a wideband frequency synthesizer and frequency generating system such as that in the SDR based WSN base station.

## 5. Conclusions

The working bandwidth and selectivity conflict with each other in traditional mixers employing LC- or Q-enhanced LC-resonant loads. In order to release this contradiction, a QSSB mixer with the proposed PNR-based load for a wide operating range and high ISRR is presented in this paper. By biasing the MOSFETs at the deep linear region periodically, the PNR is obtained to enhance the parallel LC resonator quality factor. A QSSB mixer with the PNR-based load was implemented in a TSMC 180 nm CMOS process and performed ISRR of 40–57 dB and 30–57 dB across 2.5–3 GHz and 2.3–3.2 GHz, respectively. Moreover, the noise induced by the mixer was negligible. The measurement results demonstrate that the proposed QSSB mixer not only works in a wide frequency range but also performs high ISRR. This QSSB mixer is suitable for a wideband frequency synthesizer and frequency generating system such as that in the SDR based WSN base-station.

## Figures and Tables

**Table 1:** Some typical protocols with their corresponding main characteristics.

	Operating Distance (m)	Transmit Power (dBm)	Data Rate (bps)	Frequency Band (GHz)
ZigBee	10–100	0–3	20–250 k	0.780/0.868 /0.915/2.4
Wireless HART	10–100	--	<250 k	2.4
ISA100.11a	10–100	<26	250 k	2.4
Bluetooth	1–100	0–20	1–3 M	2.4
GSM	<35 k	30–33	13 k	0.85/0.95 /1.8–1.9
WiBree	1–10	−6	1 M	2.4
RFID	<5	0	<0.4 M	0.01356/ 0.86–0.96/2.4

**Table 2:** Performance comparison with the referenced works.

	Load Type	Operating Range/GHz	ISRR/dB	Process
[6]	LC	5–6	42	0.13 μm CMOS
[7]	Q-enhanced LC	3.4/3.9/4.4/5/5.5/6.1/6.6/7.1/7.6/.2/.7/9.2/9.7/10.3	33	65 nm CMOS
[8]	LC	3.4/3.9/4.4/5/5.5/6.1/6.6/7.1/7.6	37	0.13 μm CMOS
[15]	Q-enhanced LC	3.4/4.4	30	0.18 μm CMOS
[16]	Q-enhanced LC	3.4/4.4	43	0.18 μm CMOS
[17]	LC	3.4/3.9/4.4	31	0.18 μm CMOS
[18]	LC	3.4/3.9/4.4/5/5.5/6.1/6.6/7.1/7.6/.2/.7/9.2/9.7/10.3	35	0.13 μm CMOS
[19]	LC	3.4/3.9/4.4	18–32	0.13 μm CMOS
[20]	LC	3.4/3.9/4.4/5/5.5/6.1/6.6/7.1/7.6	22–37	0.18 μm CMOS
[21]	LC	3.4/3.9/4.4/5/5.5/6.1/6.6/7.1/7.6/.2/.7/9.2/9.7/10.3	29	0.18 μm CMOS
[24]	LC	5	30	0.13 CMOS
This work	PNR with LC	2.5–3	40–57	0.18 μm CMOS
		2.3–3.2	30–57	
